# The Prevention of Ophthalmia Neonatorum

**Published:** 1902-03

**Authors:** 


### ABSTRACT.

### The Prevention of Ophthalmia Neonatorum.

Dr. Lucien Howe, of Buffalo, (*Philadelphia Med. Jour.*, January 18, 1902), in an article on the prevention of ophthalmia neonatorum, urges the enactment of laws which will make it compulsory upon the practitioner to adopt some form of prophylaxis against this disease, which is responsible for so many cases of blindness. He cites statistics by Kostling, showing that in 17,000 births where no prophylactic treatment had been employed some trace of ophthalmia developed in over 9 per cent., whereas in 24,000 children treated by the Credé method the number who developed the disease was only one-half of 1 per cent. The Credé method, however, has the disadvantage of always producing some pain and usually more or less conjunctivitis, while in a few instances it has given rise to corneal ulceration. According to the statistics of Piotrowski, in 1,030 children treated with a strong solution of boric acid and a 10 per cent. solution of protargol not a single case of ophthalmia occurred, while slight catarrhal conjunctivitis was observed in only ½ per cent. Aside from the numerous favorable reports on the value of protargol as a prophylactic against this affection by European authors, the drug is preferred for this purpose by many ophthalmologists in this country, including Drs. Alt, Peck, Cheney, Fox, Hotz, Zimmermann, Convorse and Todd.

In commenting upon Dr. Howe's paper, the *Philadelphia Medical Journal* remarks editorially:

If we cannot reach the *fons et origo* of ophthalmia neonatorum, we can at least save the offspring from a life of darkness, and protect the community from a source of burden and expense. That this can to an enormous extent be accomplished by prophylatic instillation need hardly be repeated, and its negligence constitutes a sin of omission that deserves commensurate punishment. The enactment of such a law is feasible, its interpretation obvious, and its enforcement not difficult, provided the accoucheur receives the intelligent support of an intelligently instructed community.

